# A RRAM-Based True Random Number Generator with 2T1R Architecture for Hardware Security Applications

**DOI:** 10.3390/mi14061213

**Published:** 2023-06-08

**Authors:** Bo Peng, Qiqiao Wu, Zhongqiang Wang, Jianguo Yang

**Affiliations:** 1 Key Laboratory of UV Light-Emitting Materials and Technology of Ministry of Education, Northeast Normal University, Changchun 130024, China; pengb806@nenu.edu.cn; 2 School of Microelectronics, University of Science and Technology of China, Hefei 230026, China; wuqiqiao@mail.ustc.edu.cn; 3 Key Laboratory of Microelectronic Devices Integrated Technology, Institute of Microelectronics of Chinese Academy of Sciences, Beijing 100029, China; 4 Research Center for Intelligent Computing Hardware, Zhejiang Lab, Hangzhou 311121, China

**Keywords:** RRAM, TRNG, 2T1R, error bit correction

## Abstract

Resistance random access memory (RRAM) based true random number generator (TRNG) has great potential to be applied to hardware security owing to its intrinsic switching variability. Especially the high resistance state (HRS) variation is usually taken as the entropy source of RRAM-based TRNG. However, the small HRS variation of RRAM may be introduced owing to fabrication process fluctuations, which may lead to error bits and be vulnerable to noise interference. In this work, we propose an RRAM-based TRNG with a 2T1R architecture scheme, which can effectively distinguish the resistance values of HRS with an accuracy of 1.5 kΩ. As a result, the error bits can be corrected to a certain extent while the noise is suppressed. Finally, a 2T1R RRAM-based TRNG macro is simulated and verified using the 28 nm CMOS process, which suggests its potential for hardware security applications.

## 1. Introduction

With the arrival of the Internet of Things (IoT) era, information security is becoming a great concern in universal mobile terminal devices, such as household electric appliances, CCD cameras, and online banking machines. Hence, cryptography is becoming more important, particularly the generation of secure and reliable keys. To enable a terminal device to generate and store a secure and reliable key, two conditions are usually required: (i) The unpredictability of the key generated by the entropy source, and (ii) the entropy source of randomness can be sufficiently isolated from all deterministic influences [1]. A true random number generator (TRNG) can satisfy the above two conditions due to its specific randomness and independent, statistically uniform physical entropy source [2].

So far, resistance random access memory (RRAM)-based TRNGs have been widely developed because of the natural randomness of RRAM in the resistive switching process [3,4,5,6,7,8]. Some such TRNGs are achieved through the set time variation of RRAM [9], stochastic current fluctuations [10], or high resistance state (HRS) variation of RRAM [11]. For example, Gao et al. used a pseudo-forming technique to achieve excellent robustness of TRNG [12]. Yuan et al. explored a TRNG-based Cu-doped TiO_2−x_ nanoscale memristor [13]. Song et al. optimized random telegraph noise characteristics of TRNG [14]. Taneja et al. designed an SRAM architecture with in-memory generation of both dynamic and multi-bit static entropy [15]. Baturone et al. propose a circuit named multibit-RO-PUF-TRNG, which offers the advantages of unifying PUF and TRNG in the same design [16]. Stochastic HRS variation is the most common method used to implement TRNG. For instance, the HRS variability of RRAM was utilized to generate reconfigurable random numbers in Chen’s work [17]. Liu et al. explored the generation of random numbers based on the large variability in HRS on 1T1R array architecture [18]. However, small HRS variation also exists owing to fluctuations in the RRAM fabrication process. This could impact the randomness of the RRAM-based TRNG, leading to error bits in the output. Additionally, with the small HRS variation condition, RRAM-based TRNG outputs are also vulnerable to noise or power supply voltage fluctuation. Therefore, the 1T1R architecture is not universally applicable to RRAM-based TRNGs, considering the diverse HRS variation conditions.

To solve this problem, we propose an RRAM-based TRNG scheme with a 2T1R architecture. This scheme can correct the error bit of TRNG outputs and suppress noise to some extent. First, the concept of 1T1R RRAM-based TRNG and its disadvantages induced by the small HRS variation are described. Second, the 2T1R RRAM-based TRNG scheme is proposed, and its advantages are presented. Finally, the randomness of the RRAM-based TRNG with 2T1R architecture is verified by the National Institute of Standards and Technology (NIST) test.

## 2. Proposed TRNG Scheme

### 2.1. Concept of RRAM-Based TRNG and Disadvantages of 1T1R Architecture

To illustrate the electrical characteristics and HRS variation of RRAM in this work, we refer to the RRAM Verilog-A model [19,20]. Besides describing the switching process of RRAM, this model also considers the stochastic property brought by the gap size of conductive filaments of RRAM and temperature. On the one hand, the main stochastic switching mechanisms are as follows: (i) The randomness of the ion migration process in the reset process, (ii) the variation of gap size, considering the multiple conductive filaments of RRAM. On the other hand, temperature can also strongly affect the growth rate and the amplitude of the conductive filament, resulting in some switching variation.

**Figure 1:**
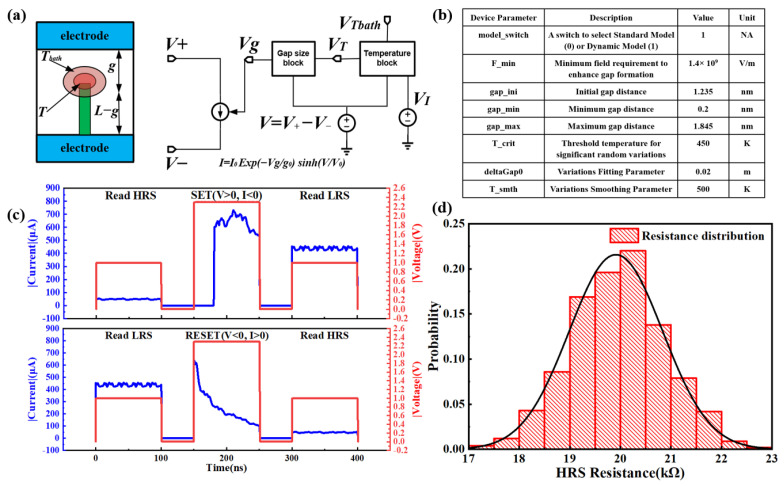
(**a**) SPICE compact model of RRAM with switching variation. (**b**) Main parameters of the SPICE compact model of RRAM. (**c**) Simulated pulse operation of SET and RESET processes. (**d**) Simulated HRS distribution of RRAM with a median value of 20 kΩ.

Based on these two physical factors, Wong’s group proposed the SPICE compact model of RRAM with switching variation [20], as shown in Figure 1a. Therefore, the simulation results of the RRAM model can guarantee the HRS variation in the switching process. To intuitively display the characteristics of this model, Figure 1b shows the main parameters employed in our simulation. By using these main physical parameters, the transient resistive switching process of RRAM is simulated, as shown in Figure 1c. It can be seen that the RRAM cell shows bipolar switching, in which the HRS can be SET to the LRS under positive voltage while the RESET process occurs under negative bias. By checking the read current before and after programming operation, the resistance values of HRS and LRS are around 20 and 2 kΩ, respectively. Furthermore, by repeating the transient simulation for 100k cycles, the HRS distribution of RRAM with a median value of 20 kΩ is obtained due to the stochastic property of the SPICE compact model, as shown in Figure 1d. The HRS of RRAM is distributed in the range from 17 to 23 kΩ, indicating a small HRS variation. The Verilog-A model and HRS variation of RRAM with a median value of 20 kΩ are utilized for the design and simulation of 1T1R and 2T1R TRNG macro circuits.

**Figure 2:**
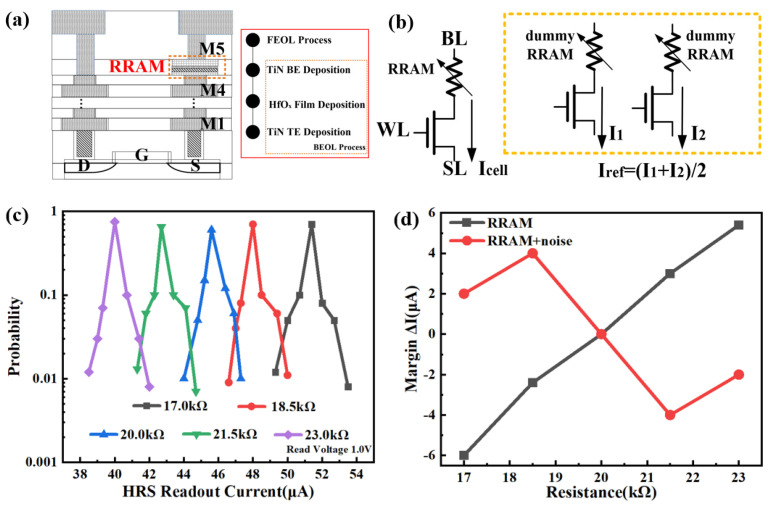
(**a**) Structural diagram of the 1T1R; the inset shows the BEOL process of the RRAM. (**b**) Schematic view of the 1T1R RRAM architecture. BL, bit line; WL, word line; SL, source line. (**c**) Simulated readout current distribution of the HRS with steps of 1.5 kΩ. The current is collected using a read pulse of 1.0 V. (**d**) Simulated readout current margins of different resistances under the presence or absence of noise conditions.

The RRAM cells of the 1T1R architecture can be integrated between the metal layers of M4 and M5 in the 28 nm logic process, as shown in Figure 2a. The box with a dashed orange line shows the structure diagram of a single HfO_x_-based RRAM device, which has a metal-resistive film-metal structure. The resistive HfO_x_ layer is sandwiched by the two TiN electrodes. After the front-end-of-line (FEOL) process of transistor fabrication, the RRAM device is fabricated with the back-end-of-line (BEOL) process [21,22], as shown in the inset of Figure 2a. The resistive HfO_x_ layer is deposited on the TiN bottom electrode by atomic layer deposition (ALD). The top electrode TiN is deposited by electron beam evaporation.

Before discussing the shortcomings of the 1T1R TRNG macro, it is necessary to describe the operation mode of the TRNG with 1T1R architecture. Figure 2b shows the schematic view of the 1T1R architecture in the TRNG macro, which includes one 1T1R cell and two 1T1R dummy cells. Three steps are required to operate the TRNG. First, in the reset operation, the word line is turned on, while voltage bias is applied to the source line, and the bit line is grounded. At this instant, the entropy source RRAM of TRNG is written as HRS, in which process the resistance value of HRS is random between the different rows and columns because the HRS of RRAM usually has a variation from device to device [23]. Second, in the readout operation, a voltage of 1.0 V is applied to the bit line, and the word line is turned on, in which process the current flowing through the source line is the readout current I_cell_ of the entropy source RRAM of TRNG. Additionally, the two 1T1R dummy cells are used to generate a reference current I_ref_, which is the average current flowing through these two 1T1R dummy cells. In fact, the HRS of the entropy source RRAM of TRNG varies with external physical factors, such as switching process and temperature. Therefore, it is necessary to utilize two 1T1R dummy cells as the reference cell. Finally, one bit of TRNG output is generated by comparing the current in the source line with the reference current. The above steps clearly explain the concept of RRAM-based TRNG with 1T1R architecture.

To show the disadvantage of the 1T1R RRAM-based TRNG, we use the above-mentioned HRS data of RRAM from 17 to 23 kΩ in steps of 1.5 kΩ. It is obvious that the reference cell should be 20 kΩ. Based on 10k Monte-Carlo simulations during the readout phase, Figure 2c shows the simulated readout current distribution of the HRS with the 1T1R architecture. Two kinds of current variations are included in the readout current distribution of Figure 2c. One is the current variation from NMOS transistor process deviation, which is introduced in the 10k Monte-Carlo simulations during the readout phase. Another one is the HRS variation from the stochastic property of the SPICE compact model, which is related to the stochastic switching factors, such as ion migration process, gap size, and temperature. The cell readout current distribution of the HRS with an accuracy of 1.5 kΩ presents the overlapping phenomenon, which means that the TRNG output bits could suffer error bits. On the other hand, we define that the current readout margin is the difference between the reference current I_ref_ and the cell readout current I_cell_, namely, ΔI = I_ref_ − I_cell_. Under the no-noise conditions, assuming 20 kΩ as the resistance value of the reference cell, the simulated readout margin of 17 or 18.5 kΩ RRAM should be negative, which is derived from the readout current of the 20 kΩ RRAM minus the readout current of the 17 or 18.5 kΩ RRAM. However, once ±5% fluctuation noise of the 1.0 V readout voltage is applied on the bit line, simulated readout margin value of the 17 or 18.5 kΩ RRAM becomes positive, which means that the TRNG outputs occur incorrect readout, as shown in Figure 2d. The simulated readout margin of the 21.5 or 23 kΩ RRAM also follows the same principle. In order to improve the reading margin of the HRS with an accuracy of 1.5 kΩ and restrain the impact of noise, the TRNG macro circuit with 2T1R architecture is proposed in this work.

### 2.2. The 2T1R RRAM-Based TRNG Scheme and Its Advantages

**Figure 3:**
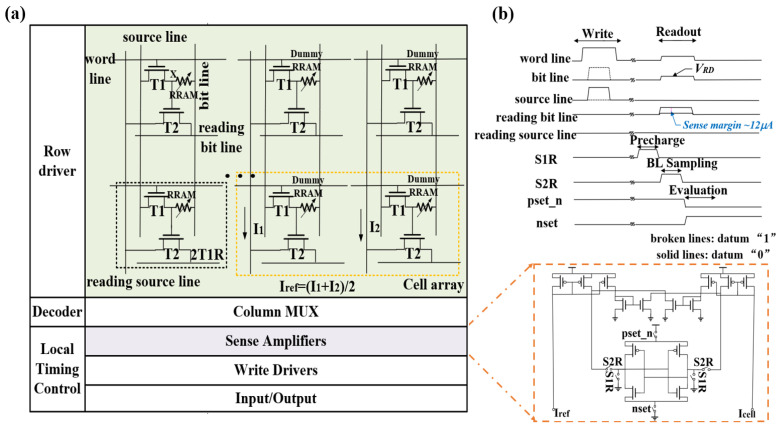
(**a**) Circuit structure of 2T1R RRAM macro; the inset shows the current-mode sense amplifier. (**b**) Operation sequence for the 2T1R RRAM-based TRNG scheme.

In the 1T1R RRAM-based TRNG, the small HRS variation of RRAM is taken as the entropy source of the TRNG, which easily generates the error bits in the TRNG macro and is also susceptible to noise. Thus, this phenomenon may generate more “1”s or “0”s, thereby affecting the randomness of RRAM-based TRNG. To solve these problems, the 2T1R RRAM-based TRNG is proposed, which adds one more transistor as a gain cell, as shown in Figure 3a. The 2T1R architecture can expand the readout margin of RRAM-based TRNG, solving the problem of error bits. Furthermore, due to the increase of the readout margin, the impact of noise or power supply voltage fluctuation can be suppressed in the 2T1R RRAM-based TRNG to a certain extent.

The operation mode of the TRNG with 2T1R architecture and its superiority will be described subsequently. An operation sequence for the 2T1R RRAM-based TRNG scheme is shown in Figure 3b. First, in the writing stage, a transistor T1 is turned on while the write pulse is applied to the source line for the datum “0”. At this moment, the entropy source RRAM of TRNG is written as the HRS, in which process the resistance value of HRS is unpredictable between different rows and columns. Second, in the readout stage, the pulses around 1.0 V are applied to the word line and the bit line, while the readout pulse is applied to the reading bit line. Considering the transistor T2 is used as a gain cell, the larger readout currents I_cell_ of the entropy source RRAM of TRNG flow out through the reading source line. For the same reason as with the 1T1R dummy cell, it is necessary to use the two-column 2T1R dummy cells as the reference cell. The reference current I_ref_ in the 2T1R RRAM-based TRNG equals the average current flowing through the two 2T1R dummy cells. Finally, in the evaluation stage, the “pset_n” and “nset” switches of the current-mode sense amplifiers are turned on. Then, one bit of TRNG output is generated by comparing the current in the reading source line with the reference current.

However, to ensure the correctness of the written data for the 2T1R RRAM-based TRNG, the verify operation is added after the write operation, that is, the readout operation. If the read data are the same as the estimated data, which means the verification is correct, the pulse sequence continues to write the next storage cell. If the verification is wrong, the pulse sequence mode continues the write operation until the verification is correct. Herein, it is noted that the precharge phase and bit line sampling phase of the current-mode sense amplifier can be operated simultaneously with the readout phase of the 2T1R cell array. The above discussion clearly describes the concept of RRAM-based TRNG with 2T1R architecture.

**Figure 4:**
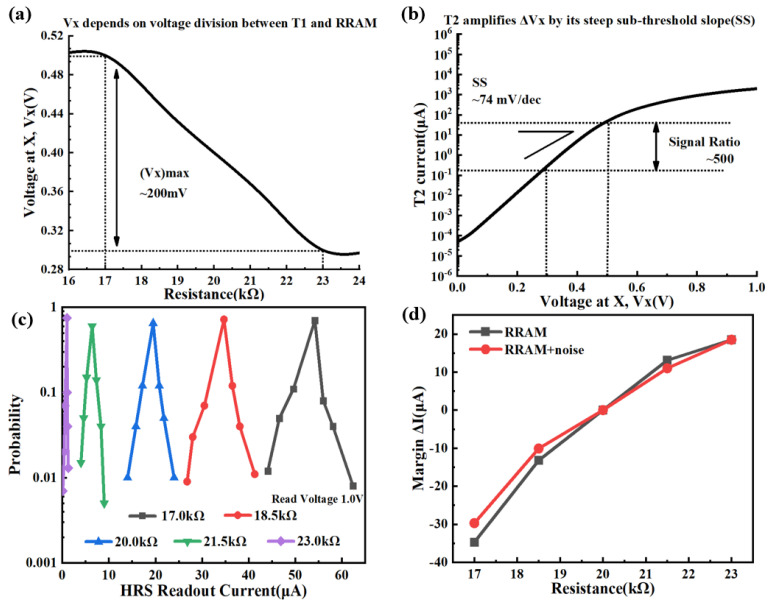
(**a**) Simulated voltage V_x_ versus HRS of RRAM. (**b**) Simulated transistor T2 current versus V_x_ at 17 and 23 kΩ; (**c**) Simulated readout current distribution of the HRS with steps of 1.5 kΩ; The current is collected using a read pulse of 1.0 V. (**d**) Simulated readout current margin of different resistances under the presence or absence of noise conditions.

To illustrate the advantage of the RRAM-based TRNG with the 2T1R architecture, its readout principle is described below, and its simulation results are compared with those of the 1T1R RRAM-based TRNG. The above-mentioned HRS data from 17 to 23 kΩ in steps of 1.5 kΩ are employed. Figure 4a shows the simulated variation of the voltage V_x_ of the gate electrode of the gain transistor T2 in the readout phase as the HRS changes from 17 to 23 kΩ. Herein, the voltage V_x_ depends on the voltage division between transistor T1 and RRAM. There is a large variation of the voltage V_x_ of the gate electrode of gain transistor T2, which is around 200 mV. The voltage variation ΔV_x_ of the gate electrode is amplified by the gain transistor T2, which is operated at near-threshold-voltage (NVT) to leverage its steep sub-threshold slope (SS) to amplify the resistance ratio, as shown in Figure 4b. This readout method corresponds to transferring the readout current from the linear variation at the 1T1R cell to the exponential variation at the 2T1R cell, which expands the readout margin of a small HRS variation of RRAM [24]. Next, the advantage of the RRAM-based TRNG with the 2T1R architecture is illustrated. Based on 10k Monte-Carlo simulations during the readout phase, Figure 4c shows the simulated readout current distribution of the HRS in steps of 1.5 kΩ. The principle of two kinds of current variations is the same as Figure 2c. As can be seen from the results presented in Figure 4c, the 2T1R architecture can effectively distinguish the HRS of RRAM with an accuracy of 1.5 kΩ. There is no overlapping part for readout, and the one bit of TRNG output is a correct bit, which ensures the randomness of the TRNG output. In terms of noise suppression, we also apply ±5% fluctuation noise of readout voltage on the bit line, during which process 20 kΩ is also chosen as the resistance value of the reference cell. As shown in Figure 4d, compared with the no-noise conditions, the simulated readout margin only slightly decreases without a reversal phenomenon, which indicates that the TRNG with 2T1R architecture has a certain ability to suppress noise. Here, it should be noted that due to the fluctuation of conductive filaments is much larger than that of random telegraph noise (RTN) [14], therefore, the impact of RTN on the reliability of the TRNG can be ignored. In the meantime, compared with the 1T1R architecture, the readout current margin of the 2T1R RRAM-based TRNG is expanded five times. To sum up, RRAM-based TRNG with the 2T1R architecture solves the problems of small readout margin and inability to suppress noise, which ensures the unpredictability of the TRNG outputs.

### 2.3. Randomness Verification of the 2T1R RRAM-Based TRNG

**Figure 5:**
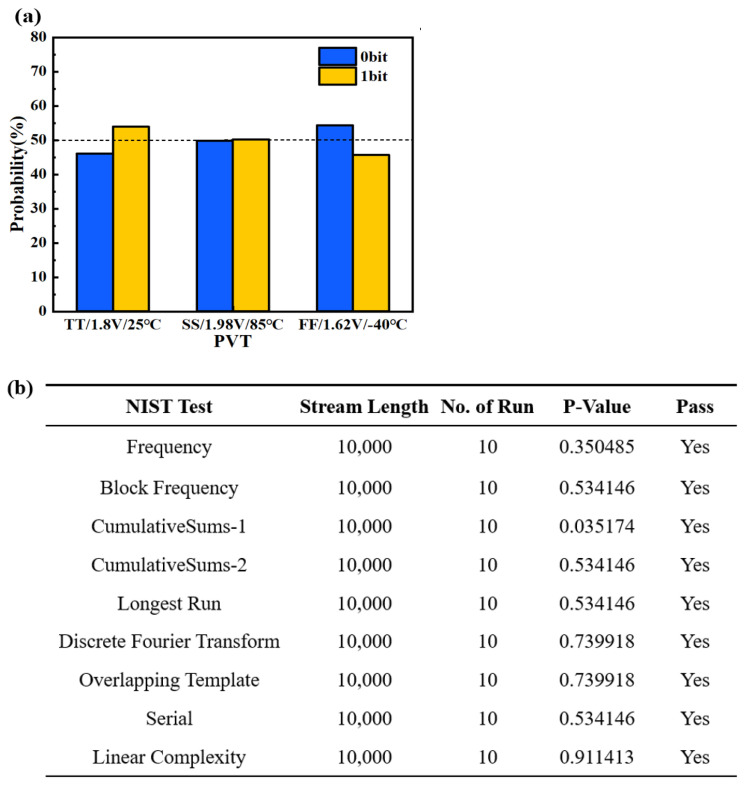

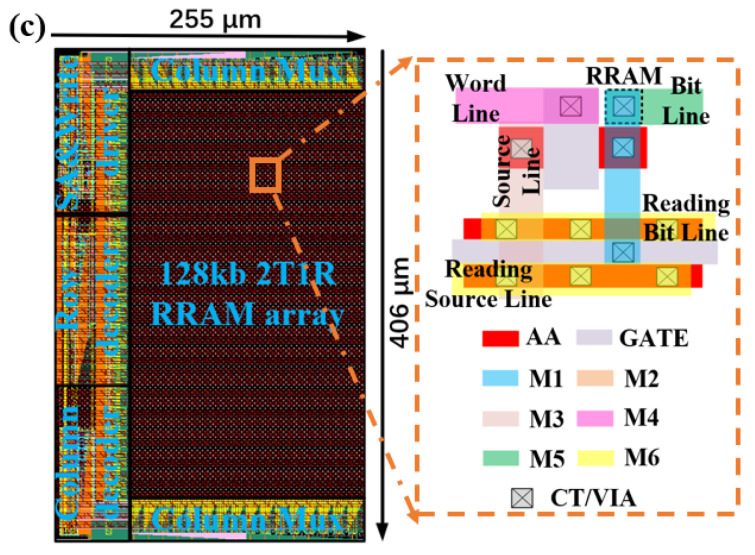
(**a**) Simulated proportion of “0” bit or “1” bit in a sample of about 100 k bits. (**b**) NIST test of the 2T1R TRNG outputs. (**c**) Layout of the 128 Kb 2T1R RRAM; the inset shows the layout of a single 2T1R cell array, where “AA” means the active area of the transistor, and “GATE” means the gate electrode of the transistor.

To verify the randomness characteristic of the 2T1R RRAM-based TRNG, the above-mentioned simulated HRS distribution data ranging from 17 to 23 kΩ are still used. As described in Section 2.1, we have obtained the simulated HRS distribution data with a median value of 20 kΩ based on the Verilog-A model, which consists of a series of random data of HRS. Assuming 20 kΩ as the resistance value of the reference cell, we can obtain a series of “0” bit or “1” bit of the TRNG output data. This means that the RRAM of each row and each column outputs one accurate bit in the 2T1R array. After a simulated sample of collecting about 100 k bits, the proportion of “0” bit or “1” bit converges to nearly 50% under various process corners and voltages and temperature (PVT) simulated conditions, as shown in Figure 5a. By collecting about 100 k bits as a simulated sample, 2T1R TRNG output bits can also pass the NIST test, including the frequency test, the cumulative sums test, the runs test, the discrete Fourier transform (spectral) test, etc., as shown in Figure 5b. These tests verify the randomness of the 2T1R RRAM-TRNG output bits. Additionally, the endurance of the RRAM device is generally larger than 10^3^ [12,14]. Considering the 128 Kb RRAM array, the TRNG can generate around 10^8^ random numbers. Even considering a 50% yield rate, the TRNG can generate around 5 × 10^7^ random numbers, which meets the requirements of the TRNG.

Figure 5c shows the layout of the 128 Kb 2T1R RRAM-based TRNG macro, which has an area of 255 μm × 406 μm and is composed of parts including the 2T1R cell array, the current-mode sense amplifier, the column mux circuit, the write drive circuit, the row drive circuit, the local timing control circuit, etc. The inset shows the layout of a single 2T1R cell array. Finally, the power consumption and readout response speed of the whole 2T1R RRAM-based TRNG macro circuit are also simulated under various PVT conditions, as shown in Table 1. The overall average power consumption of the whole 2T1R RRAM-based TRNG macro circuit is around 18 μW. The average readout response speed of the current-mode sense amplifier is about 15 ns. Table 2 shows the comparison of this work with state-of-the-art TRNGs [3,4,5]. The advantages of the proposed TRNG macro are as follows: Error bit correction and noise suppression. In addition, the proposed TRNG macro also can work under larger PVT variation conditions.

## 3. Conclusions

In this study, a 2T1R RRAM-based TRNG scheme is proposed for hardware security applications. This scheme exhibits the advantages of error bit correction and noise suppression. In terms of error bit correction, the 2T1R architecture can effectively distinguish the small HRS variation of RRAM with an accuracy of 1.5 kΩ. In terms of noise suppression, at least ±5% fluctuation noise of readout voltage can be effectively suppressed. Meanwhile, the randomness of the 2T1R RRAM-based TRNG macro is verified by the NIST test. These advantages of a 2T1R RRAM-based TRNG suggest its great potential for hardware security applications in the future.

## Figures and Tables

**Table 1:** Simulated power and readout speed of the whole 2T1R RRAM-TRNG macro.

PVT	Power Consumption	Response Speed
TT/1.8 V/25 °C	17.53 μW	15 ns
SS/1.8 V/−40 °C	15.61 μW	10 ns
FF/1.8 V/85 °C	18.89 μW	13 ns
TT/1.98 V/25 °C	19.91 μW	17 ns
SS/1.98 V/−40°C	17.79 μW	16 ns
FF/1.98 V/85 °C	21.41 μW	18 ns
TT/1.62 V/25 °C	15.25 μW	15 ns
SS/1.62 V/−40 °C	13.52 μW	10 ns
FF/1.62 V/85 °C	16.46 μW	12 ns

**Table 2:** Comparison of proposed TRNG with state-of-the-art TRNGs.

TRNG	This Work	Ref. [12]	Ref. [13]	Ref. [14]
Entropy source	RRAM	RRAM	Chaotic map	RTN
Unified functions	TRNG	PUF + TRNG	TRNG	TRNG
Error bit correction	Yes	No	No	No
Suppression noise	Yes	Yes	No	Yes
Readout circuit	Yes	Yes	Yes	Yes
Supply voltage (V)	1.62–1.98	0.9–1.3	0.8–1.0	1.0–1.5
Temperature (°C)	−40–85	−55–125	25	25
TRNG energy (fj/bit)	1750	1983	NA	NA
Max throughout (Mbps)	10	1.5	12.5	10^−5^

## Data Availability

The data that support the findings of this study are available from the corresponding author upon request.
